# Implementation of specific strength training among industrial laboratory technicians: long-term effects on back, neck and upper extremity pain

**DOI:** 10.1186/1471-2474-14-287

**Published:** 2013-10-09

**Authors:** Mogens Theisen Pedersen, Christoffer H Andersen, Mette K Zebis, Gisela Sjøgaard, Lars L Andersen

**Affiliations:** 1 Department of Nutrition, Exercise and Sports, University of Copenhagen, Nørre Allé 51, DK-2200 Copenhagen N, Denmark; 2 National Research Centre for the Working Environment, LersøParkallè 105, DK-2100 Copenhagen Ø, Denmark; 3 Arthroscopic Centre Amager & Gait Analysis Laboratory, Copenhagen University Hospital, Amager-Hvidovre, Kettegaard Alle 30, DK-2650 Hvidovre, Denmark; 4 Institute of Sport Sciences and Clinical Biomechanics, University of Southern Denmark, Campusvej 55, DK-5320 Odense M, Denmark

## Abstract

**Background:**

Previous studies have shown positive effects of physical exercise at the workplace on musculoskeletal disorders. However, long-term adherence remains a challenge. The present study evaluates long-term adherence and effects of a workplace strength training intervention on back, neck and upper extremity pain among laboratory technicians.

**Methods:**

Cluster-randomized controlled trial involving 537 industrial laboratory technicians. Subjects were randomized at the cluster level to one of two groups: training group 1 (TG1, n = 282) performing supervised strength training from February to June 2009 (round one) or training group 2 (TG2, n = 255) performing supervised strength training from August to December 2009 (round two). The outcome measures were changes in self-reported pain intensity (0–9) in the back, neck and upper extremity as well as Disability of the Arm, Shoulder and Hand (DASH, 0–100).

**Results:**

Regular adherence, defined as at least one training session per week, was achieved by around 85% in both groups in the supervised training periods. In the intention-to-treat analyses there were significant *group by time* effects for pain in the neck, right shoulder, right hand and lower back and DASH - resulting in significant reductions in pain (mean 0.3 to 0.5) and DASH (mean 3.9) in the scheduled training group compared to the reference group. For TG1 there were no significant changes in pain in round two, i.e. they maintained the pain reduction achieved in round one. Subgroup analyses among those with severe pain (> = 3 on a scale of 0–9) showed a significant *group by time* effect for pain in the neck, right shoulder, upper back and lower back. For these subgroups the pain reduction in response to training ranged from 1.1 to 1.8.

**Conclusions:**

Specific strength training at the workplace can lead to significant long-term reductions in spinal and upper extremity pain and DASH. The pain reductions achieved during the intensive training phase with supervision appears to be maintained a half year later.

## Background

Musculoskeletal disorders (MSD) among the working population constitutes a challenge to the public health systems in many countries [1]. The prevalence of MSD is high in certain occupations. Laboratory technicians – e.g. doing pipetting work – show high prevalence of neck pain, shoulder pain, elbow pain and hand pain [2,3]. Pain in shoulder and hand is associated with the duration of the pipetting work and neck pain is associated with the amount of fixed working postures when pipetting [2,3]. In addition sedentary work has been associated with an excess risk of low back pain [4]. Reduced workability and absence from work are common consequences of MSD [5]. Thus, initiatives to reduce the burden of MSD are needed.

Recent reviews have shown that physical training –especially strength training – at the work place can reduce neck pain, but the evidence of an effect on pain in shoulder and low back among sedentary workers is limited, and some studies show no effect [6,7]. Furthermore, the majority of this type of research has focused on office workers. We recently showed that specific resistance training and all-round physical exercise had positive effects on musculoskeletal pain symptoms in the neck, low back, right elbow and right hand among office workers [8]. Even brief exercise sessions of a few minutes each day for ten weeks can provide significant reductions of perceived neck/shoulder pain [9], decreased frequency of headaches [10] and increased pressure pain threshold [11] in office workers. Upper back pain is seldom reported in training intervention studies and to our knowledge nobody has documented an effect of training on upper back pain. The results from previous training intervention studies makes it relevant to examine the effect of strength training on pain symptoms in several regions of the upper body among laboratory technicians doing repetitive but more forceful work compared to office workers. A small study among laboratory technicians found significant reduction in back and neck pain as well as improved muscle functional performance in response to strength training with kettlebells [12,13].

In spite of many studies showing positive results of strength training for MSD, adherence to the training interventions especially in a long-term perspective remains a challenge. High dropout rates are often reported during the initial weeks, suggesting that motivation of the employees rapidly declines. We have recently performed a workplace training intervention study among laboratory technicians which included a waiting-list group that was engaged in strength training after half a year. It can be speculated that motivation and concomitant adherence and effects would be lower when such a waiting list group finally starts their training. We previously reported results on neck, shoulder and forearm pain from the first 20 weeks of this intervention [14,15]. The present study describes the effect of 12 months of the strength training intervention including the training period for the waiting group and follow up for the original training group and considering more body regions.

## Methods

### Study design

**Figure 1:**
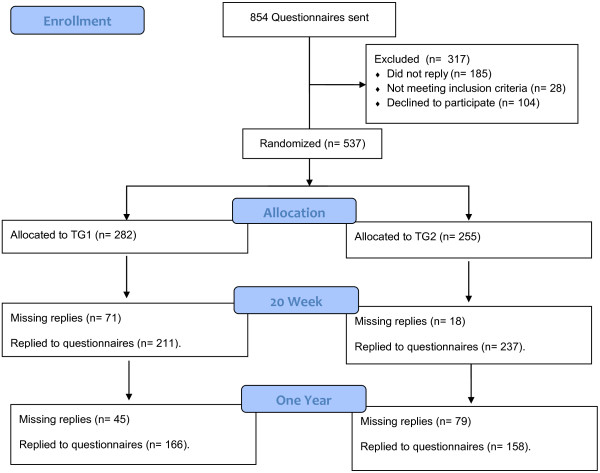
Flow of participants throughout the intervention.

This study reports the results of a one year cluster randomized controlled trial with two strength training intervention periods (January-June/June-January) among industrial laboratory technicians in Copenhagen, Denmark. Materials and methods have been described in more details elsewhere [14,16]. The participants recruited were working-age (i.e. 18–67 years) laboratory technicians with repetitive work tasks. We sent an internet-based questionnaire to 854 prospective participants of which 669 replied (Figure 1). 104 declined to participate or did not reply to the question concerning participation. Exclusion criteria – which led to exclusion of 28 participants – were pregnancy and serious health conditions such as previous trauma or injuries, life-threatening diseases and cardiovascular disease. Thereby 537 participants were included in the study and randomly assigned at the cluster level to either the first training group (TG1, n = 282) or the second training group (TG2, n = 255). At 20 weeks follow-up 211 (75%) participants from TG1 and 237 (93%) from TG2 replied the questionnaires. At the one year follow-up 166 from TG1 and 158 participants from TG2 replied the questionnaires, corresponding to 59% and 62% of the numbers at baseline.

All participants gave their written informed consent to participate in this study, which conformed to the Declaration of Helsinki and was approved by the “Ethical Committee C of Region Hovedstaden” (HC2008103). Trial registration: http://clinicaltrials.gov (NCT01071980).

### Description of intervention

The intervention took place over one year with questionnaires sent out in January 2009, June 2009, and January 2010. TG1 performed specific strength training for the shoulder, neck and arm with dumbbells (wrist extension, shoulder lateral raise, shoulder front raise, shoulder shrugs, reverse flies) for 20 min three times per week for 20 weeks. The exercises are illustrated in Zebis et al. [14] at http://www.biomedcentral.com/1471-2474/12/205. Training loads were progressively increased from moderate loadings of 15–20 repetitions maximum (RM – i.e. the number of repetitions that could be performed to momentary fatigue) during the initial weeks to relatively heavier loadings of 8–12 RM during the final weeks. The exercises were performed slowly in a controlled manner. Experienced instructors supervised every other training session to ensure correct technique and sufficient progression of the training. The participants in the training group were instructed to register the training load for each training session in a personal logbook during the 20-week intervention period.

We tried to improve compliance by placing the training facilities close to the working place to reduce transportation time and distance as a barrier for not training.

Adherence was quantified from questionnaire replies on training frequency at follow-up. The reply options given in the questionnaire were: “2-3 times per week”, “1-2 times per week”, “1 time per week”, ″2-3 times per month”, “never” and “withdrew from intervention”. Regular adherence was defined as participating at least once a week during the intervention [17].

For the whole year of intervention subjects in TG1 and TG2 were advised to continue their normal physical activity as usual regardless of the training interventions. After 20 weeks TG2 was offered the same training as TG1 did the first 20 weeks (including supervision every other training session and using logbooks) for half a year until January 2010. Participants in TG1 were allowed to continue training until 2010 but without supervision or any form of guidance. TG1 was not allowed to train, when TG2 had supervised training. However, they were allowed to train during working hours as previously and the management was positive to this continued training. Since this training was unscheduled and on their own hand – probably on an irregular basis – the amount of training was not recorded.

### Primary outcome measures

Pain intensity during the last 7 days was reported according to the Nordic questionnaire on trouble (ache, pain, or discomfort) in the neck, shoulders, elbows, hands, upper back and lower back [18]. The intensity of pain was rated subjectively on a scale ranging from 0–9 in the questionnaire, where 0 indicated “no pain at all” and 9 indicated “worst imaginable pain”. The following questions were asked: “What degree of pain or discomfort have you experienced in [body part] during the last 7 days ?” with [body part] replaced first by neck, then by the right and left shoulder, then by right and left elbow, then by right and left hand then by then upper back, and then by lower back. For baseline measurements subjects also rated pain intensity during the last three months.

Cases (neck/R-shoulder/L-Shoulder/R-elbow/L-Elbow/R-hand/L-hand/upper back/lower back) were defined as subjects with a pain rating of 3 or more on the 0–9 scale. A cut-point of 3 for cases was chosen based on a previous study by Kaergaard and coworkers [19] showing that pain intensities at 3 is a relevant cut-point for increased prevalence of clinical findings. Thus, pain intensities of 0–2 can be considered minor or no pain from a clinical perspective. Therefore subjects with pain intensities of less than 3 were defined as non-cases.

Participants rated *work disability* at baseline and follow-up by the work module of the Disability of the Arm Shoulder and Hand (DASH) questionnaire, which has previously been validated among industrial workers [20]: “In the past week did you have any difficulty:” (1) “using your usual technique for your work?”, (2) “doing your usual work because of arm, shoulder or hand pain?”, (3) “doing your work as well as you would like?” and (4) spending your usual amount of time doing your work?”. Participants replied on a 5-point Likert scale from ‘no difficulty’ to ‘unable’. The work disability score was normalised on a scale of 0 to100, where 100 represents the highest level of disability [21].

### Statistical analyses

We performed analyses in accordance with the intention-to-treat principle, and used repeated measures analysis of variance to determine between-group differences in pain intensity for each body region. We did not impute missing data as all methods of data imputation have limitations [22]. Instead we used the MIXED procedure of SAS which inherently accounts for missing data. Group (TG1 and TG2) and time (baseline, 1. follow-up and 2. Follow-up) and group by time interaction was entered in the model as fixed effects. Participant and cluster were entered as random effects. Analyses were adjusted for baseline pain intensity. We used the SAS statistical software for the analyses (SAS institute, Cary, NC, version 9.2), and accepted an alpha level of 5% as statistically significant. These analyses were also performed for cases at baseline (pain intensity of 3 or more on the 0–9 scale).

In case of a significant intervention effect post hoc test were included in the ANOVA to look for changes between and within groups from baseline to 20 week follow up and from 20 week follow up to one year follow up. Post hoc chi-square tests were used to look for differences in clinical significant changes (2 or more on the 0–9 scale) between groups. All analyses were controlled for gender. We report baseline results as means (SD) and between-group differences at each time-point as least square means (SE).

The vast majority of the study group was right handed and the work tasks were often defined as being performed by the right hand independent on handedness. Therefore we did not report results for dominant versus non-dominant limbs but for right and left.

## Results

Table 1 shows baseline characteristics for TG1and TG2. Except for gender there were no significant differences between groups. Pain in L-shoulder, L-elbow and L-hand were very low and with no significant changes during the intervention (data not shown).

**Table 1:** Baseline characteristics

	**First training group (N = 255)**	**Second training group (N = 282)**
**Age (years)**	42 (10)	42 (11)
**Height (cm)**	170 (8)	168 (8)
**Weight (kg)**	73 (14)	70 (14)
**Body mass index (kgm**^**-2**^**)**	25 (5)	28 (4)
**Women%**	80%	89%
**DASH**	18,4 (21,5)	15,1 (21)
**Pain (the last three months)**		
Neck	2,9 (2,2)	2,7 (2,2)
Right shoulder	2,5 (2,3)	2,3 (2,3)
Right Elbow	1,3 (2,3)	1,0 (2,0)
Right Hand	1,9 (2,3)	1,8 (2,3)
Upper back	1,9 (2,1)	1,9 (2.2)
Lower Back	2,3 (2,3)	2,5 (2,4)
**Number of cases at baseline**		
Neck	95	78
Right shoulder	76	70
Right Elbow	38	25
Right Hand	55	40
Upper back	55	47
Lower Back	70	67

Mean (SD) or percent distribution of the participants in the first training group (TG1) and the second training group (TG2).

Adherence:

87% of the participants in TG1 trained regularly during the first 20 weeks and 67% reported a training frequency of 2–3 times a week. 85% of the participants in TG2 trained regularly from June 09 to January 10 and 60% reported a training frequency of 2–3 times a week.

**Figure 2:**
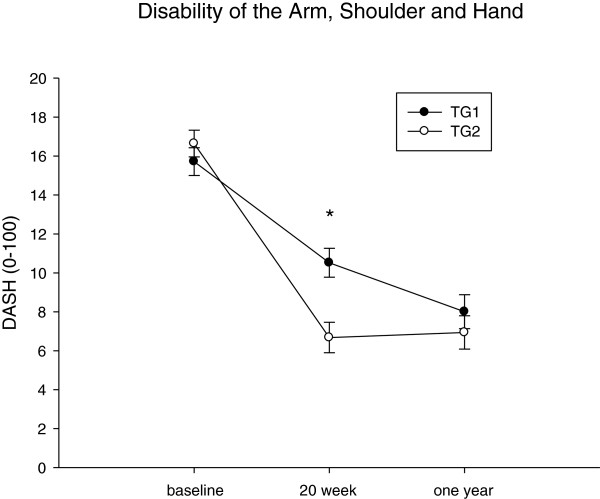
**Disability of the Arm, Shoulder and Hand (DASH) at baseline, at 20 weeks follow-up and at one-year follow up.** TG1 = first training group; TG2 = second training group. Error bars = SE. * significant time by group effect.

**Figure 3:**
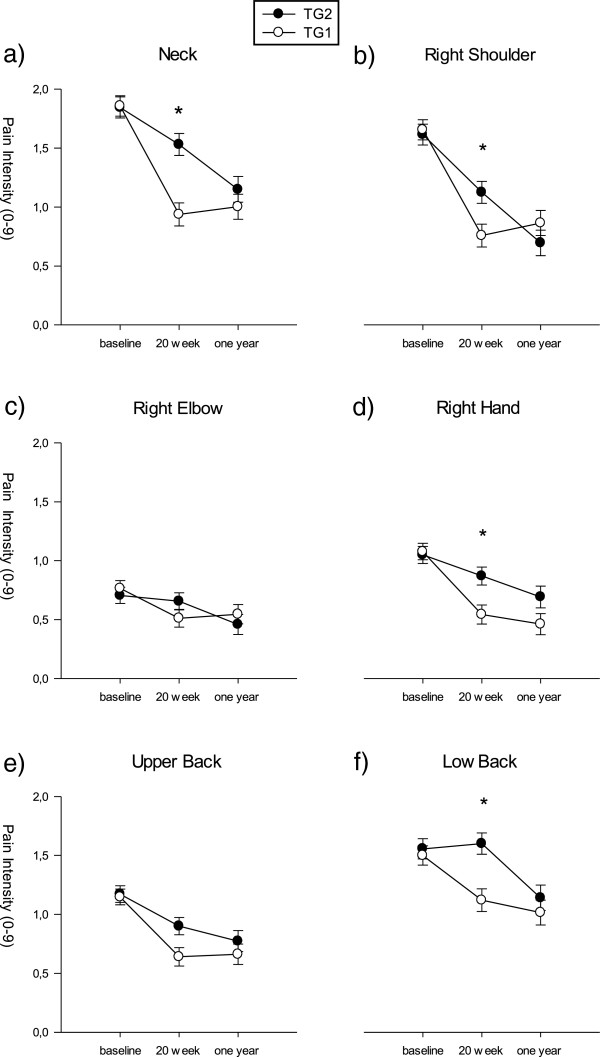
**Pain intensity the last 7 days in six regions at baseline, at 20 weeks follow-up and at one-year follow up.** TG1 = first training group; TG2 = second training group; **a-f** = graph number. Error bars = SE. * significant time by group effect.

Musculoskeletal pain and disability:

Intention-to-treat analysis across the one year intervention showed a significant time effect for pain in neck, R-shoulder, R-elbow, R-hand, upper back and lower back and DASH to decrease (p < 0,01-0,0001). This resulted in significantly lower values for pain in all these regions and for DASH at one year follow up compared to baseline in both groups (Figures 2, 3).

There was a significant group by time effect for pain in the neck, R-shoulder, R-hand and lower back and for DASH (p < 0.001 for Neck and DASH; p < 0.05 for R-shoulder, R-hand and low back). Changes in pain in these regions and changes in DASH followed the same pattern: From identical baseline values, pain and DASH in TG1declined more than in TG2 resulting in significant differences between groups at 4 months follow-up (Figures 2, 3(a,b,d,f)). Between 4 months and one year follow-up pain and DASH declined in TG2 with no significant change in the TG1 resulting in identical values for both groups at one year follow up (Figures 2, 3(a,b,d,f), Table 2).

**Table 2:** Changes within groups (Intention-to-treat)

	**Group**	**Time**		**Differences of least squares means (SE)**	**P**
**Neck**	0	1	2	0.31(0.12)	0.008
	0	1	3	0.70(0.13)	<0.001
	0	2	3	0.38(0.13)	0.004
	1	1	2	0.92(0.12)	<0.001
	1	1	3	0.85(0.13)	<0.001
	1	2	3	-0.06(0.14)	0.633
**Right shoulder**	0	1	2	0.49(0.12)	<0.001
	0	1	3	0.92(0.13)	<0.001
	0	2	3	0.43(0.14)	0.002
	1	1	2	0.90(0.12)	<0.001
	1	1	3	0.79(0.13)	<0.001
	1	2	3	-0.11(0.14)	0.441
**Right hand**	0	1	2	0.18(0.10)	0.076
	0	1	3	0.36(0.11)	0.002
	0	2	3	0.18(0.12)	0.123
	1	1	2	0.53(0.10)	<0.001
	1	1	3	0.62(0.11)	<0.001
	1	2	3	0.08(0.12)	0.480
**Low back**	0	1	2	-0.04(0.12)	0.717
	0	1	3	0.42(0.14)	0.002
	0	2	3	0.46(0.14)	<0.001
	1	1	2	0.38(0.12)	0.002
	1	1	3	0.48(0.13)	<0.001
	1	2	3	0.11(0.14)	0.452
**Dash**	0	1	2	5.19(0.95)	<0.001
	0	1	3	7.70(1.06)	<0.001
	0	2	3	2.51(1.08)	0.021
	1	1	2	9.96(0.97)	<0.001
	1	1	3	9.70(1.03)	<0.001
	1	2	3	-0.26(1.10)	0.812

Time wise Changes within groups (0 = second intervention group, 1 = first intervention group ) for Pain and disability index (DASH) and pain intensity (0–9 scale) the last 7 days in the Neck and Right Shoulder, Right Hand and Low Back (Time: 1 = baseline, 2 = 20 weeks follow-up and 3 = one-year follow up). P-value for post hoc t-tests.

**Figure 4:**
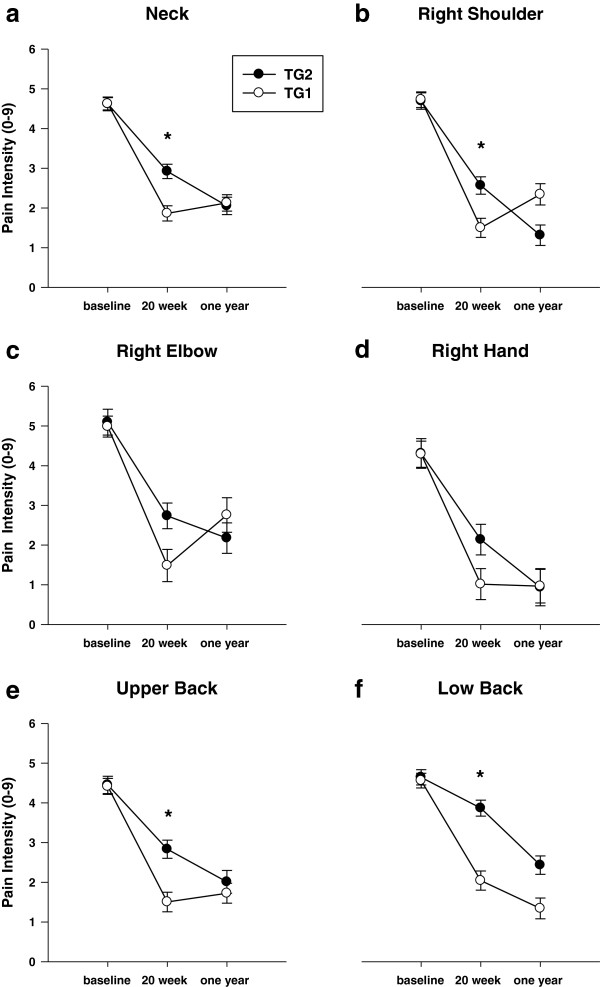
**Cases.** Pain intensity the last 7 days in six regions at baseline, at 20 weeks follow-up and at one-year follow up. TG1 = first training group; TG2 = second training group; **a-f** = graph number. Error bars = SE. * significant time by group effect.

Secondary analysis looking at cases only showed significant intervention effects for pain in the neck, R-shoulder, upper back and low back (p < 0.001). The time wise changes were larger than in the intention to treat analyses but followed the same pattern with the greatest decline in pain in TG1 between baseline and 20 week follow up, and a decline in TG2 with no significant change in the first training group (or for the right shoulder an increase in pain) between 20 weeks and one year follow up (Figure 4(a,b,e,f), Table 3).

**Table 3:** Changes within groups (cases only)

	**Group**	**Time**		**Differences of least squares means**	**P**
**Neck**	0	1	2	1.70(0.24)	<0.001
	0	1	3	2.57(0.27)	<0.001
	0	2	3	0.87(0.27)	0.002
	1	1	2	2.76(0.24)	<0.001
	1	1	3	2.50(0.25)	<0.001
	1	2	3	-0.26(0.27)	0.330
**Right shoulder**	0	1	2	2.13(0.29)	<0.001
	0	1	3	3.38(0.32)	<0.001
	0	2	3	1.25(0.32)	<0.001
	1	1	2	3.23(0.30)	<0.001
	1	1	3	2.38(0.32)	<0.001
	1	2	3	-0.84(0.34)	0.014
**Upper back**	0	1	2	1.62(0.30)	<0.001
	0	1	3	2.44(0.35)	<0.001
	0	2	3	0.82(0.35)	0.020
	1	1	2	2.91(0.30)	<0.001
	1	1	3	2.69(0.30)	<0.001
	1	2	3	-0.22(0.33)	0.508
**Low back**	0	1	2	0.78(0.27)	0.005
	0	1	3	2.21(0.30)	<0.001
	0	2	3	1.43(0.30)	<0.001
	1	1	2	2.52(0.30)	<0.001
	1	1	3	3.22(0.32)	<0.001
	1	2	3	0.70(0.35)	0.048

Time wise Changes within groups (0 = second intervention group, 1 = first intervention group ) for Pain and disability index (DASH) and pain intensity (0–9 scale) the last 7 days in the Neck, Right Shoulder, Upper Back and Low Back (Time: 1 = baseline, 2 = 20 weeks follow-up and 3 = one-year follow up). P-value for post hoc t-tests.

**Figure 5:**
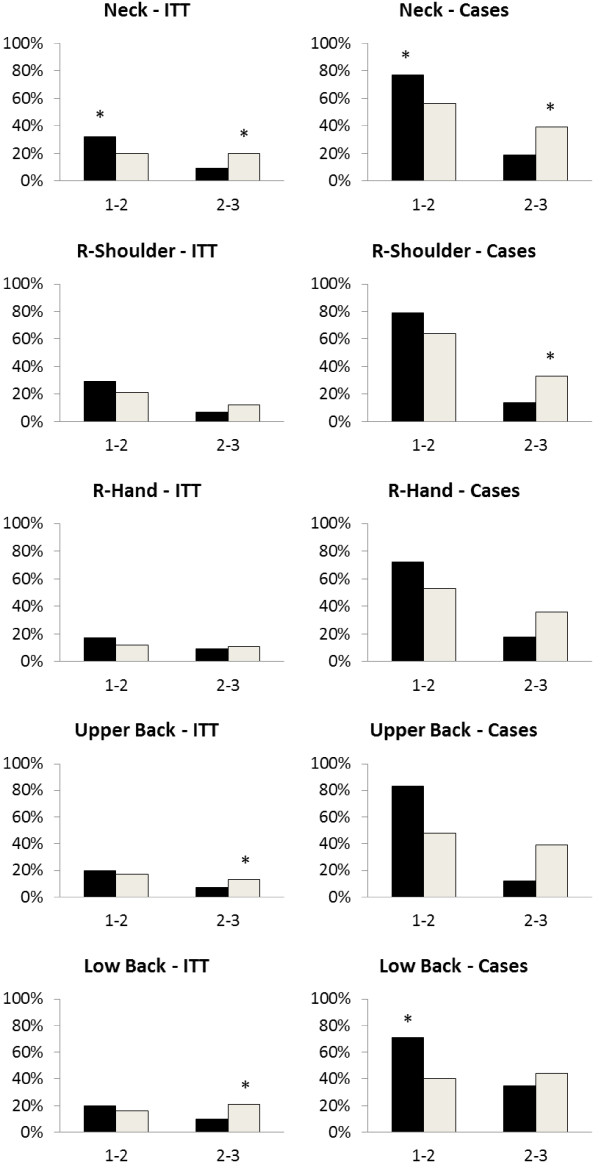
**Percentage of subjects with reductions in pain intensity of 2 or more on a 0–9 scale in five different body regions.** Intention-to-treat (ITT) analyses among all participants (left column). Participants with pain > = 3 at baseline (right column). On the x-axis 1–2 denotes changes during the first intervention round where the first training group (black bars) performed specific strength training, and 2–3 denotes changes during the second intervention round where the second training group (white columns) performed specific strength training. * significant differences between changes in TG1 and TG2 (chi-square test).

Figure 5 shows the result of post hoc analyses of the distribution of the percentage of subjects in each training group with clinically significant reductions in pain (2 or more on 0–9 scale) between rounds.

## Discussion

The main finding of this one year intervention study is that the largest effects in terms of decrease in musculoskeletal pain and disability was attained during the periods of organized and supervised strength training, irrespective of the time of the season. The study had an effect on pain in Neck, R-shoulder, R-hand and upper and lower back as well as on DASH as a result of the supervised training intervention period. Another important finding is that having participated in such strength training for 20 weeks initially in the study a long-term effect was attained, i.e. the decrease of musculoskeletal pain and disability was maintained at one-year follow-up.

According to questionnaires regular adherence was achieved by 87% of the participants in TG1during the first 20 weeks and by 85% of the participants in TG2 from June 09 to January 10. At the one year follow-up around 40% did not reply to the questionnaire with no significant difference between TG1 (41%) and TG2(38%), so although being a waiting list group motivation and concomitant adherence was not lower in TG2 compared to TG1. However, this also highlights the difficulties in obtaining high follow-up response rates in long-term research studies. To account for the missing replies we used a statistical procedure which inherently accounts for missing values, i.e. the mixed procedure.

There was a significant time effect for pain in all regions and DASH showing significantly lower values after one year follow-up compared to baseline (Figures 2, 3). Thus for TG1 self-administered training did not reduce pain further but pain was kept on the new reduced level probably due to training as without training we would expect an increase in pain in the autumn, due to normal seasonal variation [23] herhaps due to physical activity normally being highest in summer and less in the winter. Accordingly pain and disability index decreased in TG2 in the spring due seasonal variation (Figures 1, 2).

This study adds to the earlier published results on pain in shoulder, neck and forearm as well as DASH [14,15] by documenting an effect also on R-hand and upper and lower back as well as the long-term maintenance of the positive effects. Beaton et al. showed in a case series design (uncontrolled longitudinal study) that DASH was responsive to treatment, i.e. DASH scores decreased with 12 weeks of treatment in 200 patients with wrist/hand or shoulder disability. The mean overall differences in changes in pain between groups (Figure 3) were up to 0.7 which is less than the minimal perceptible change on an individual basis [24] and much less than the minimal value of 2 for a clinical significant individual change [24]. For cases only the mean changes were higher (1.06 to 1.82; Figure 4). Small effect sizes like these are normal with clinical trials and it may be more informative to examine the distribution of responses between treatment groups [24]. Therefore we looked at the number of subjects having clinical significant reductions (i.e. a decrease of at least 2 on a ten-point scale) in pain (Figure 5) and found significant differences in the distribution of changes between groups with the highest reductions in the actual training groups (Figure 5). For the neck these changes were significant between groups after both intervention periods and for cases as well as for all subjects. For right shoulder and upper and lower back there were significant differences between changes in groups for some combinations of periods/ITT/Cases suggesting clinically significant intervention effects. For right hand there were no significant differences in the clinically significant changes between groups.

These distributions of differences in changes between groups presents additional evidence for the effect of physical training on neck pain as reported in systematic reviews [6,7] as well as the evidence of a moderate effect of training on low back pain [6,25-27]. It also supports a few high quality studies showing effectiveness of training on shoulder symptoms [28,29] and combined neck/shoulder symptoms [9,22]. Our study was unique in reporting an effect of strength training on upper back pain among cases. To our knowledge this has not been reported in previous training intervention studies. The shoulder girdle attaches by muscles to the scapula and the back of the thoracic rib cage. These upper back muscles are prone to developing irritation that can be painful. In clinical practice pain complaints from the neck, the shoulder girdle, and part of the shoulder go together [30]. Neck, shoulder, and upper back muscles are all involved during repetitive movements/activity of the arms with a common effect on all three regions. Importantly this study report results on laboratory technicians as opposed to other occupational groups reported in the above mentioned studies.

The small but significant effect on R-hand pain may be due to a change in central pain perception, which is known to be altered in chronic pain conditions [31]. A change in neck pain could result in beneficial changes in overall pain perception and a decreased pain sensitization. A previous study showed central adaptations of pain perception in response to neck/shoulder rehabilitation, i.e. pressure pain threshold increased also in other non-trained parts of the body [11].

The limited evidence for a clinical effect on R-hand pain and no evidence for an effect on elbow pain could be due to low power as a result of low pain at baseline compared to pain in Neck, Shoulder, Low back and Upper back. Recently we showed an effect of a one year training intervention on pain in right elbow and right hand [8] among office workers. Increasing the training periods for TG1 + TG2 might have resulted in more significant results for pain in R-elbow and R-hand.

Participants in TG1 were allowed to continue training until January 2010 in spite that their supervised training period ended medio 2009, but they did not receive any guidance from the training instructors and were not provided with new training diaries. As a result TG1 may have performed very limited amount of training in the autumn. An important novel finding in this study was that the effect of training on pain in TG1 lasted half a year after the intensive supervised period. This is in line with a previous study on office workers with chronic neck muscle pain, where pain relief as a result of strength training was prolonged and unchanged 10 weeks after the intervention [32]. We therefore cannot rule out if the prolonged pain reduction in TG1 in the autumn was a result of continued training or a prolonged effect of the training in the first intervention period or a combination on both. Lack of supervision may partly explain the lack of further reduction in pain in TG1 in the autumn as supervision seems to be a precondition for a training intervention effect [6].

The significant intervention effect on DASH although small points to an increased ability to work as a result of strength training and ads to the positive results of the training intervention.

As reported earlier employees with higher pain levels were more interested in participating than those with lower pain levels [14] suggesting that pain per se was no hindrance for participating.

The intention-to-treat analysis, showed only small average changes in pain, but including all employees instead of only subjects with pain [33-37] reveals the overall impact of the intervention and thus the public health perspective.

The present study has both limitations and strengths. Using a cluster RCT design with a high number of participants increases statistical power of the study. Further, to increase external validity and generalizability we included both public and private sector companies. A limitation is the possible influence of placebo in behavioral interventions. Further, a limitation is that the first intervention group was not monitored for training adherence during the second period where they acted as controls.

## Conclusions

Specific strength training at the workplace can lead to significant long-term reductions in spinal and upper extremity pain and DASH. The pain reductions achieved during the intensive training phase with supervision appears to be maintained a half year later, i.e. follow up with self-administered training can keep pain on a low level but does not result in further pain reduction.

## Competing interests

The authors declare that they have no competing interests.

## Authors’ contributions

MTP, CHA, MKZ, GS and LLA were responsible for the research design. MTP drafted the paper, and all co-authors made significant contributions to drafting and to the protocol. All authors have read and approved the final manuscript.

## Pre-publication history

The pre-publication history for this paper can be accessed here:

http://www.biomedcentral.com/1471-2474/14/287/prepub

## References

[B1] Punnett L Wegman DH Work-related musculoskeletal disorders: the epidemiologic evidence and the debate J Electromyogr Kinesiol 2004 14 13 23 10.1016/j.jelekin.2003.09.015 14759746

[B2] Bjorksten MG Almby B Jansson ES Hand and shoulder ailments among laboratory technicians using modern plunger-operated pipettes Appl Ergon 1994 25 88 94 10.1016/0003-6870(94)90069-8 15676954

[B3] David G Buckle P A questionnaire survey of the ergonomic problems associated with pipettes and their usage with specific reference to work-related upper limb disorders Appl Ergon 1997 28 257 262 10.1016/S0003-6870(97)00002-1 9414365

[B4] Thorbjornsson CB Alfredsson L Fredriksson K Michelsen H Punnett L Vingard E Physical and psychosocial factors related to low back pain during a 24-year period. A nested case–control analysis Spine 2000 25 369 374 10.1097/00007632-200002010-00019 10703112

[B5] Andersen LL Mortensen OS Hansen JV Burr H A prospective cohort study on severe pain as a risk factor for long-term sickness absence in blue- and white-collar workers Occup Environ Med 2011 68 590 592 10.1136/oem.2010.056259 21071754

[B6] Coury HJ Moreira RF Dias NB Evaluation of the effectiveness of workplace exercise in controlling neck, shoulder and low back pain: a systematic review Rev Bras Fisioter 2009 13 461 479 10.1590/S1413-35552009000600002

[B7] Sihawong R Janwantanakul P Sitthipornvorakul E Pensri P Exercise therapy for office workers with nonspecific neck pain: a systematic review J Manipulative Physiol Ther 2011 34 62 71 10.1016/j.jmpt.2010.11.005 21237409

[B8] Andersen LL Christensen KB Holtermann A Poulsen OM Sjogaard G Pedersen MT Effect of physical exercise interventions on musculoskeletal pain in all body regions among office workers: a one-year randomized controlled trial Man Ther 2010 15 100 104 10.1016/j.math.2009.08.004 19716742

[B9] Andersen LL Saervoll CA Mortensen OS Poulsen OM Hannerz H Zebis MK Effectiveness of small daily amounts of progressive resistance training for frequent neck/shoulder pain: randomised controlled trial Pain 2011 152 440 446 10.1016/j.pain.2010.11.016 21177034

[B10] Andersen LL Mortensen OS Zebis MK Jensen RH Poulsen OM Effect of brief daily exercise on headache among adults–secondary analysis of a randomized controlled trial Scand J Work Environ Health 2011 37 547 550 10.5271/sjweh.3170 21617837

[B11] Andersen LL Andersen CH Sundstrup E Jakobsen MD Mortensen OS Zebis MK Central adaptation of pain perception in response to rehabilitation of musculoskeletal pain: randomized controlled trial Pain Physician 2012 15 385 394 22996850

[B12] Jay K Frisch D Hansen K Zebis MK Andersen CH Mortensen OS Kettlebell training for musculoskeletal and cardiovascular health: a randomized controlled trial Scand J Work Environ Health 2011 37 196 203 10.5271/sjweh.3136 21107513

[B13] Jay K Jakobsen MD Sundstrup E Skotte JH Jorgensen MB Andersen CH Effects of kettlebell training on postural coordination and jump performance: A randomized controlled trial J Strength Cond Res 2013 27 1202 1209 10.1519/JSC.0b013e318267a1aa 22843044

[B14] Zebis MK Andersen LL Pedersen MT Mortensen P Andersen CH Pedersen MM Implementation of neck/shoulder exercises for pain relief among industrial workers: a randomized controlled trial BMC Musculoskelet Disord 2011 12 205 10.1186/1471-2474-12-205 21936939 PMC3188479

[B15] Andersen LL Jakobsen MD Pedersen MT Mortensen OS Sjogaard G Zebis MK Effect of specific resistance training on forearm pain and work disability in industrial technicians: cluster randomised controlled trial BMJ Open 2012 2 e000412 10.1136/bmjopen-2011-000412 22331386 PMC3282287

[B16] Andersen LL Zebis MK Pedersen MT Roessler KK Andersen CH Pedersen MM Protocol for work place adjusted intelligent physical exercise reducing musculoskeletal pain in shoulder and neck (VIMS): a cluster randomized controlled trial BMC Musculoskelet Disord 2010 11 173 10.1186/1471-2474-11-173 20687940 PMC2921353

[B17] Andersen LL Jorgensen MB Blangsted AK Pedersen MT Hansen EA Sjogaard G A randomized controlled intervention trial to relieve and prevent neck/shoulder pain Med Sci Sports Exerc 2008 40 983 990 10.1249/MSS.0b013e3181676640 18461010

[B18] Kuorinka I Jonsson B Kilbom A Vinterberg H Biering-Sorensen F Andersson G Standardised Nordic questionnaires for the analysis of musculoskeletal symptoms Appl Ergon 1987 18 233 237 10.1016/0003-6870(87)90010-X 15676628

[B19] Kaergaard A Andersen JH Rasmussen K Mikkelsen S Identification of neck-shoulder disorders in a 1 year follow-up study. Validation of a questionnaire-based method Pain 2000 86 305 310 10.1016/S0304-3959(00)00261-X 10812260

[B20] Kitis A Celik E Aslan UB Zencir M DASH questionnaire for the analysis of musculoskeletal symptoms in industry workers: a validity and reliability study Appl Ergon 2009 40 251 255 10.1016/j.apergo.2008.04.005 18555973

[B21] Beaton DE Katz JN Fossel AH Wright JG Tarasuk V Bombardier C Measuring the whole or the parts? Validity, reliability, and responsiveness of the Disabilities of the Arm, Shoulder and Hand outcome measure in different regions of the upper extremity J Hand Ther 2001 14 128 146 10.1016/S0894-1130(01)80043-0 11382253

[B22] Moher D Hopewell S Schulz KF Montori V Gotzsche PC Devereaux PJ CONSORT 2010 explanation and elaboration: updated guidelines for reporting parallel group randomised trials BMJ 2010 340 c869 10.1136/bmj.c869 20332511 PMC2844943

[B23] Takala EP Viikari-Juntura E Moneta GB Saarenmaa K Kaivanto K Seasonal variation in neck and shoulder symptoms Scand J Work Environ Health 1992 18 257 261 10.5271/sjweh.1580 1411369

[B24] Dworkin RH Turk DC McDermott MP Peirce-Sandner S Burke LB Cowan P Interpreting the clinical importance of group differences in chronic pain clinical trials: IMMPACT recommendations Pain 2009 146 238 244 10.1016/j.pain.2009.08.019 19836888

[B25] Burton AK Balague F Cardon G Eriksen HR Henrotin Y Lahad A How to prevent low back pain Best Pract Res Clin Rheumatol 2005 19 541 555 10.1016/j.berh.2005.03.001 15949775

[B26] Hayden JA Van Tulder MW Tomlinson G Systematic review: strategies for using exercise therapy to improve outcomes in chronic low back pain Ann Intern Med 2005 142 776 785 10.7326/0003-4819-142-9-200505030-00014 15867410

[B27] Hayden JA Van Tulder MW Malmivaara AV Koes BW Meta-analysis: exercise therapy for nonspecific low back pain Ann Intern Med 2005 142 765 775 10.7326/0003-4819-142-9-200505030-00013 15867409

[B28] Blangsted AK Sogaard K Hansen EA Hannerz H Sjogaard G One-year randomized controlled trial with different physical-activity programs to reduce musculoskeletal symptoms in the neck and shoulders among office workers Scand J Work Environ Health 2008 34 55 65 10.5271/sjweh.1192 18427699

[B29] Ludewig PM Borstad JD Effects of a home exercise programme on shoulder pain and functional status in construction workers Occup Environ Med 2003 60 841 849 10.1136/oem.60.11.841 14573714 PMC1740414

[B30] Andersen JH Kaergaard A Mikkelsen S Jensen UF Frost P Bonde JP Risk factors in the onset of neck/shoulder pain in a prospective study of workers in industrial and service companies Occup Environ Med 2003 60 649 654 10.1136/oem.60.9.649 12937185 PMC1740607

[B31] Arendt-Nielsen L Graven-Nielsen T Muscle pain: sensory implications and interaction with motor control Clin J Pain 2008 24 291 298 10.1097/AJP.0b013e31815b608f 18427227

[B32] Andersen LL Kjaer M Sogaard K Hansen L Kryger AI Sjogaard G Effect of two contrasting types of physical exercise on chronic neck muscle pain Arthritis Rheum 2008 59 84 91 10.1002/art.23256 18163419

[B33] Randlov A Ostergaard M Manniche C Kryger P Jordan A Heegaard S Intensive dynamic training for females with chronic neck/shoulder pain. A randomized controlled trial Clin Rehabil 1998 12 200 210 10.1191/026921598666881319 9688035

[B34] Viljanen M Malmivaara A Uitti J Rinne M Palmroos P Laippala P Effectiveness of dynamic muscle training, relaxation training, or ordinary activity for chronic neck pain: randomised controlled trial BMJ 2003 327 475 12946968 10.1136/bmj.327.7413.475 PMC188429

[B35] Waling K Sundelin G Ahlgren C Jarvholm B Perceived pain before and after three exercise programs–a controlled clinical trial of women with work-related trapezius myalgia Pain 2000 85 201 207 10.1016/S0304-3959(99)00265-1 10692619

[B36] Ylinen J Takala EP Nykanen M Hakkinen A Malkia E Pohjolainen T Active neck muscle training in the treatment of chronic neck pain in women: a randomized controlled trial JAMA 2003 289 2509 2516 10.1001/jama.289.19.2509 12759322

[B37] Ylinen JJ Hakkinen AH Takala EP Nykanen MJ Kautiainen HJ Malkia EA Effects of neck muscle training in women with chronic neck pain: one-year follow-up study J Strength Cond Res 2006 20 6 13 16503693 10.1519/R-17274.1

